# A Machine Learning Algorithm for Predicting the Risk of Developing to M1b Stage of Patients With Germ Cell Testicular Cancer

**DOI:** 10.3389/fpubh.2022.916513

**Published:** 2022-06-29

**Authors:** Li Ding, Kun Wang, Chi Zhang, Yang Zhang, Kanlirong Wang, Wang Li, Junqi Wang

**Affiliations:** ^1^Department of Urology, the Affiliated Hospital of Xuzhou Medical University, Xuzhou, China; ^2^Nanjing First Hospital, Nanjing, China

**Keywords:** machine learning algorithms, prediction model, germ cell testicular cancer, M1b stage, real-world research

## Abstract

**Objective:**

Distant metastasis other than non-regional lymph nodes and lung (i.e., M1b stage) significantly contributes to the poor survival prognosis of patients with germ cell testicular cancer (GCTC). The aim of this study was to develop a machine learning (ML) algorithm model to predict the risk of patients with GCTC developing the M1b stage, which can be used to assist in early intervention of patients.

**Methods:**

The clinical and pathological data of patients with GCTC were obtained from the Surveillance, Epidemiology, and End Results (SEER) database. Combing the patient's characteristic variables, we applied six machine learning (ML) algorithms to develop the predictive models, including logistic regression(LR), eXtreme Gradient Boosting (XGBoost), light Gradient Boosting Machine (lightGBM), random forest (RF), multilayer perceptron (MLP), and k-nearest neighbor (kNN). Model performances were evaluated by 10-fold cross-receiver operating characteristic (ROC) curves, which calculated the area under the curve (AUC) of models for predictive accuracy. A total of 54 patients from our own center (October 2006 to June 2021) were collected as the external validation cohort.

**Results:**

A total of 4,323 patients eligible for inclusion were screened for enrollment from the SEER database, of which 178 (4.12%) developing M1b stage. Multivariate logistic regression showed that lymph node dissection (LND), T stage, N stage, lung metastases, and distant lymph node metastases were the independent predictors of developing M1b stage risk. The models based on both the XGBoost and RF algorithms showed stable and efficient prediction performance in the training and external validation groups.

**Conclusion:**

S-stage is not an independent factor for predicting the risk of developing the M1b stage of patients with GCTC. The ML models based on both XGBoost and RF algorithms have high predictive effectiveness and may be used to predict the risk of developing the M1b stage of patients with GCTC, which is of promising value in clinical decision-making. Models still need to be tested with a larger sample of real-world data.

## Introduction

Testicular cancer (TC), as a rare malignant tumor of the genitourinary system, accounts for about 1% of male tumors and about 5% of urogenital tumors. In Occident, the annual rate of new cases is <1 in 10,000 (1). Despite having a relatively low overall incidence rate and a good prognosis, TC is the most common malignancy in men aged 15 to 35 years (2, 3). Germ cell testicular cancer (GCTC) is the most common kind of testicular cancer, accounting for over 95% of all testicular cancer histological types. There are two types of GCTC: seminoma and non-seminomatous germ cell tumors (NSGCTs). The former is the most common type of GCTC, accounting for about one-third of its total, and the latter includes embryonal carcinomas, yolk sac tumors, choriocarcinomas, teratomas, and mixed germ cell cancers (4). Cryptorchidism, family history, Klinefelter's syndrome, androgen insensitivity syndrome (AIS), and industrial exposure may be the major risk factors for testicular cancer (5–8). Serum levels of alphafetoprotein (AFP), human chorionic gonadotropin (hCG), and lactate dehydrogenase (LDH) should be determined before and after orchiectomy, as they can assist in diagnosis and predict prognosis. Genetic studies have shown that TC is associated with ectopic short arms of chromosome 12 (i12p) and that alterations in the P53 gene have a correlation with their occurrence (1, 9). Radical orchiectomy, together with bilateral retroperitoneal lymph node dissection, is the standard surgical management of patients with TC, and radiotherapy and/or chemotherapy is recommended for patients with advanced TC (10, 11).

Germ cell testicular cancer outward invasion includes lymph nodes, lungs, liver, brain, bones, etc. Although distant metastases are more likely to invade the lungs and distant lymph nodes for GCTC, the risk of other atypical metastases (including liver, brain, bones, and other rare organs or tissues), which account for approximately 10% of all patients, cannot be ignored (12–16). The International Germ Cell Cancer Collaborative Classification for Metastatic Testicular Cancer (IGCCCG) identifies non-pulmonary visceral metastases as a strong influence on poor prognosis in metastatic patients with TC (15). A recent study also showed that patients with liver metastases and bone metastases had a significantly poor prognosis compared to distant lymph node and lung metastases (13). Although most metastatic lesions are not palpable, if a patient has supraclavicular lymph node metastases, they may palpate a left cervical mass. Lung metastases may present with the shortness of breath or even rare hemoptysis. If a patient has extensive retroperitoneal metastases, they may present with low back pain due to organ compression. Meanwhile, brain metastases may cause headaches as well as various neurological symptoms (17). Contrast-enhanced computerized tomography (CECT) is the most sensitive method to evaluate patients with TC for tumor invasion in the chest, abdomen, and pelvis (18, 19). Although both CECT and magnetic resonance imaging (MRI) are the key image modalities for detecting brain metastases, MRI is much more sensitive than CECT, and therefore, MRI plays a major role in detecting brain metastases (20).However, imaging scans may not be effective enough in screening out patients with GCTC at high risk for developing to M1b stage. Therefore, a model to predict the risk of progression to M1b in patients with GCTC can be used for clinical applications to improve patient prognosis.

Machine learning (ML) is an advanced algorithmic model that automatically learns and improves performance by identifying complex non-linear relationships in different patterns and is considered superior to traditional algorithms (21–23). As one of the topics of artificial intelligence (AI), ML has been widely used in clinical practice, such as image recognition, complications prediction, and survival analysis (24, 25). The aim of this study was to establish and validate an ML-based model predicting the risk of progression to the M1b stage in patients with GCTC.

## Materials and Methods

### Data Collection

A retrospective cohort research approach was adopted. The information came from the SEER research database, which covers approximately 27.8% of the US population. We used I CD-O-3 site codes C62.1 and C62.9 and histological codes 9061 to 9064, 9070 to 9071, 9080 to 9085, and 9100 to 9102 to identify patients with GCTC. To develop the ideal ML model, several variables were obtained, including survival data, age, race, marital status at diagnosis, histology type, TNM stage, tumor laterality, radiotherapy documents, chemotherapy documents, LND, lymph-vascular invasion (LVI), metastatic sites, and AFP/hCG/ LDH index after orchiectomy. We evaluated the S-stage of patients based on the postoperative serum tumor marker data obtained above. An external validation set was constructed by collecting the same variables from the Affiliated Hospital of Xuzhou Medical University. The flow chart for patient selection of the SEER database is shown in Supplementary Figure 1.

### Statistical Analysis

For continuous variables, the Student's *t*-test was used for normally distributed data and the Mann–Whitney *U*-test for non-normally distributed data. The chi-square test was used to analyze categorical data. The Kaplan–Meier method was being used to determine the clinical endpoints of the patients, and the log-rank test was used to analyze them. Uni- and multivariate logistic regression analyses were used to calculate the odds ratio (OR) with 95% confidence intervals (Cis). Only two-sided *p*-value <0.05 was considered statistical significance. We used six different ML algorithms to analyze our data: LR, XGBoost, lightGBM, RF, MLP, and kNN. The model with the highest average AUC was chosen as the best algorithm. Furthermore, the ML-based model was tuned to avoid overfitting, and the accuracy of the algorithm was tested using the 10-fold cross-validation method. R 4.1.2 (https://www.r-project.org/), Python 3.10 (https://www.python.org/), and SEER^*^Stat (https://seer.cancer.gov/seers tat/) were used in this study. Detailed packages used in the development of our ML models including XGBoost 1.2.1, lightGBM 3.2.1, and sklearn 0.22.1. For the kNN classifier, the number of neighbors is set as 3. For the RF algorithm, we set the “ntree” as 100 and “mtree” as 3. To avoid overfitting and enhance interpretability, the maximum tree depth was set to 8 nodes in the XGBoost algorithm. The hidden layer sizes of MLP algorithm were (10, 10).

## Results

### Patient's Characteristics

**Figure 1:**
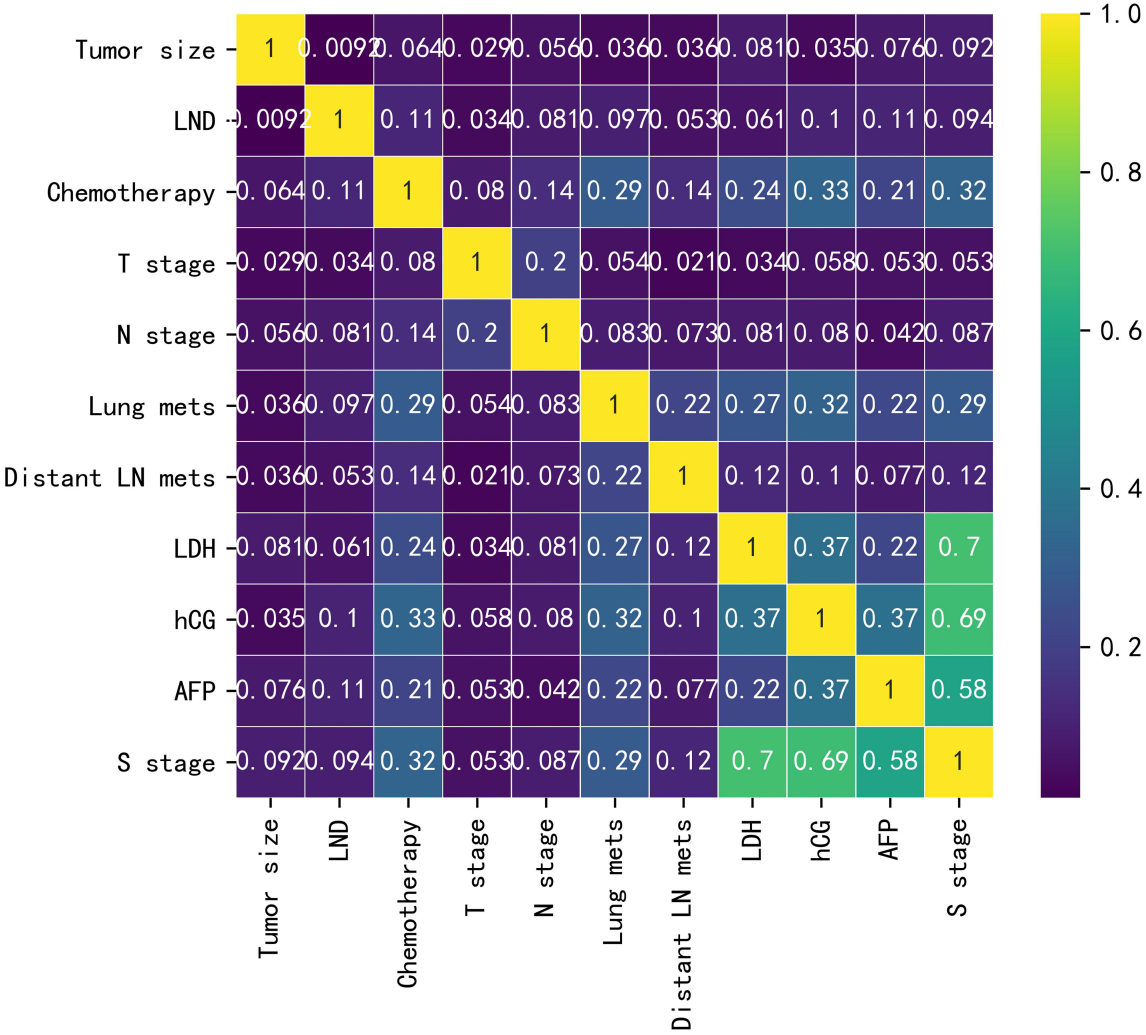
Heatmap of the correlation of patients' clinical and pathological features.

Baseline data for the training cohort and external validation cohort are listed in Supplementary Table 1. In the training cohort, the variables with *p* < 0.05 were LND, chemotherapy, T-stage, N-stage, lung metastasis, distant lymph node metastasis, LDH, hCG, AFP, and S-stage. The differences were not statistically significant in age, tumor size, race, histology type, laterality, marital status, radiotherapy, and LVI. The correlations between the variables chosen as predictors were analyzed and visualized by a heatmap using Spearman's rank correlation coefficient (Figure 1).

### Survival Analysis

**Figure 2:**
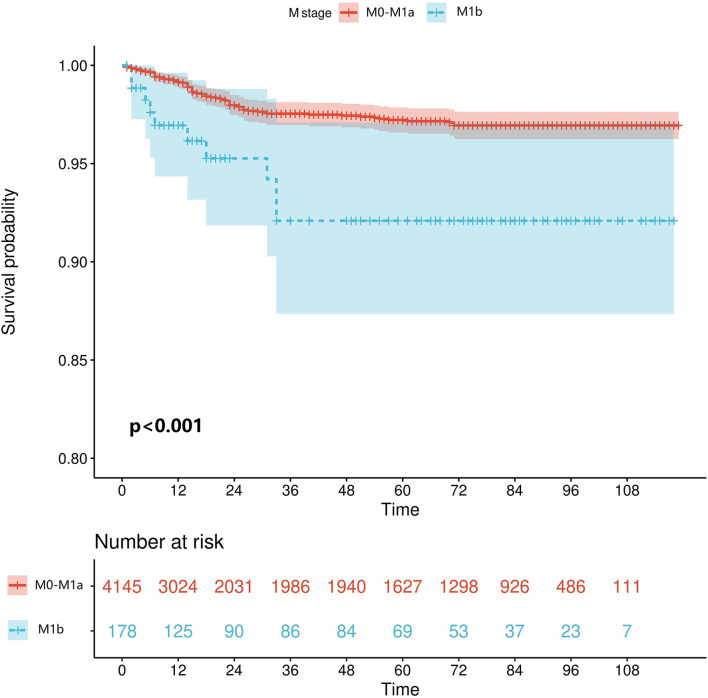
Kaplan–Meier curve of cancer-specific survival in patients with GCTC.

We retrieved patients' survival data from the SEER database, cancer-specific survival (CSS) was considered as the endpoint, and Kaplan–Meier survival analysis was used to estimate the survival. As shown in Figure 2, patients who reached the M1b stage had significantly worse CSS (*p* < 0.001).

### Univariate and Multivariate Logistic Regression Analyses

As illustrated in Table 1, in terms of univariate logistic regression analysis, LND, chemotherapy, T-stage, N-stage, lung metastasis, distant lymph node metastasis, LDH, hCG, AFP, and S-stage were all significantly associated with the occurrence of developing M1b stage in the overall population (*p* < 0.05). In multivariable logistic regression analysis (Table 2), given the high correlation between serum tumor markers and S-stage as shown by heatmap, two models were carried out to avoid collinearity. Factors with statistical significance were T-stage, N-stage, lung metastasis, and distant lymph node metastasis (*p* < 0.001) in both model 1 (included S-stage) and model 2 (included three serum tumor markers). The *p*-value of LND was 0.056 in model 1 and 0.049 in model 2. After comprehensively considering the performance of this variable in the two models, we finally incorporated it into the model algorithm of ML.

**Table 1:** Univariable logistic regression analysis of the training cohort.

**Variables**	**Level**	**Univariate OR**	**95%CI**	***p*** **-value**
Age (year)	NA	1.006	[0.993, 1.019]	0.367
Tumor size (mm)	NA	1.002	[0.999, 1.005]	0.113
Race	White	Ref		0.602
	Black	0.672	[0.211, 2.141]	0.501
	Other	1.191	[0.739, 1.919]	0.473
Histology type	Seminoma	Ref		0.139
	NGSTC	1.257	[0.928, 1.701]	
Laterality	Left	Ref		0.83
	Right	1.033	[0.765, 1.396]	
Marital status	Single	Ref		0.505
	Married	1.205	[0.881, 1.648]	0.242
	Other status	1.08	[0.596, 1.957]	0.799
LND	No/Biopsy only	Ref		<0.001
	Yes	2.309	[1.592, 3.349]	
Radiotherapy	No	Ref		0.984
	Yes	0.993	[0.501, 1.969]	
Chemotherapy	No	Ref		<0.001
	Yes	2.571	[1.854, 3.566]	
LVI	Absent	Ref		0.643
	Present	0.926	[0.668, 1.283]	
T stage	T1	Ref		<0.001
	T2	1.379	[0.973, 1.955]	0.071
	T3	6.214	[4.118, 9.377]	<0.001
	T4	10.848	[3.425, 34.362]	<0.001
N stage	N0	Ref		<0.001
	N1	5.214	[3.485, 7.801]	<0.001
	N2	4.166	[2.622, 6.620]	<0.001
	N3	9.431	[6.300, 14.119]	<0.001
Lung metastasis	No	Ref		<0.001
	Yes	4.648	[3.264, 6.620]	
Distant lymph node metastasis	No	Ref		<0.001
	Yes	9.593	[5.674, 16.218]	
LDH (U/l)	Within normal limits	Ref		0.002
	<1.5 x N	1.5	[1.008, 2.233]	0.045
	1.5–10 x N	2.109	[1.315, 3.383]	0.002
	>10 x N	2.822	[1.268, 6.283]	0.011
	Only know elevated after orchiectomy	0.914	[0.285, 2.931]	0.88
hCG (mIU/ml)	Within normal limits	Ref		<0.001
	<5,000	1.44	[0.967, 2.144]	0.072
	5,000–50,000	2.765	[1.307, 5.849]	0.008
	5,000–50,000	4.814	[2.400, 9.657]	<0.001
	Only know elevated after orchiectomy	1.926	[0.589, 6.297]	0.278
AFP (ng/ml)	Within normal limits	Ref		0.011
	<1,000	1.07	[0.714, 1.603]	0.742
	1,000–9,999	2.88	[1.546, 5.367]	0.001
	≤ 10,000	1.374	[0.327, 5.764]	0.664
S-stage	S0	Ref		<0.001
	S1	1.143	[0.756, 1.729]	0.527
	S2	1.607	[1.104, 2.338]	0.013
	S3	3.262	[1.889, 5.631]	<0.001

*OR, odds ratio; CIs, confidence intervals; NSGCT, non-seminomatous germ cell tumor; LND, lymph node dissection; LVI, lymph-vascular invasion;LDH, lactate dehydrogenase; hCG, human chorionic gonadotropin; AFP, alpha-fetoprotein; other marital status includes divorced/widowed/unknown; N indicates the upper limit of normal; serum tumor markers were determined after orchiectomy/before chemotherapy*.

**Table 2:** Multivariate logistic regression analysis of the training cohort.

**Variables**	**Level**	**Model 1**			**Model 2**		
		**Multivariate OR**	**95%CI**	***p*** **-value**	**Multivariate OR**	**95%CI**	***p*** **-value**
LND	No/Biopsy only	Ref		0.056			0.049
	Yes	1.492	[0.989, 2.250]		1.517	[1.002, 2.295]	
Chemotherapy	No	Ref		0.085			0.117
	Yes	1.397	[0.955, 2.044]		1.358	[0.926, 1.991]	
T stage	T1	Ref		<0.001			<0.001
	T2	1.053	[0.728, 1.523]		1.072	[0.74, 1.554]	
	T3	3.216	[2.054, 5.035]		3.259	[2.074, 5.121]	
	T4	5.6	[1.643, 19.090]		5.079	[1.436, 17.965]	
N stage	N0	Ref		<0.001			<0.001
	N1	4.201	[2.756, 6.404]		4.291	[2.808, 6.559]	
	N2	3.159	[1.945, 5.129]		3.288	[2.019, 5.354]	
	N3	6.148	[3.159, 1.945]		6.416	[4.138, 9.947]	
Lung metastasis	No	Ref		<0.001			0.001
	Yes	2.396	[1.538, 3.734]		2.254	[1.406, 3.613]	
Distant lymph node metastasis	No	Ref		<0.001			<0.001
	Yes	4.288	[2.335, 7.877]		4.588	[2.494, 8.441]	
LDH (U/l)	Within normal limits	/	/	/			0.697
	<1.5 x N	/	/	/	1.014	[0.644, 1.599]	
	1.5–10 x N	/	/	/	0.735	[0.404, 1.339]	
	>10 x N	/	/	/	0.976	[0.376, 2.532]	
	Only know elevated after orchiectomy	/	/	/	0.495	[0.142, 1.721]	
hCG (mIU/ml)	Within normal limits	/	/	/			0.177
	<5,000	/	/	/	1.021	[0.634, 1.645]	
	5,000–50,000	/	/	/	1.368	[0.553, 3.382]	
	5,000–50,000	/	/	/	2.873	[1.196, 6.901]	
	Only know elevated after orchiectomy	/	/	/	1.57	[0.434, 5.689]	
AFP (ng/ml)	Within normal limits	/	/	/			0.396
	<1,000	/	/	/	0.703	[0.442, 1.116]	
	1,000–9,999	/	/	/	1.143	[0.544, 2.403]	
	≤ 10,000	/	/	/	0.611	[0.123, 3.029]	
S-stage	S0	Ref		0.397	/	/	/
	S1	0.834	[0.534, 1.302]		/	/	/
	S2	0.791	[0.512, 1.221]		/	/	/
	S3	1.299	[0.678, 2.489]		/	/	/

*OR, odds ratio; Cis, confidence intervals; LND, lymph node dissection; LVI, lymph-vascular invasion;LDH, lactate dehydrogenase; hCG, human chorionic gonadotropin; AFP, alpha-fetoprotein; N indicates the upper limit of normal; serum tumor markers were determined after orchiectomy/before chemotherapy*.

### Performance of ML Algorithms

**Figure 3:**
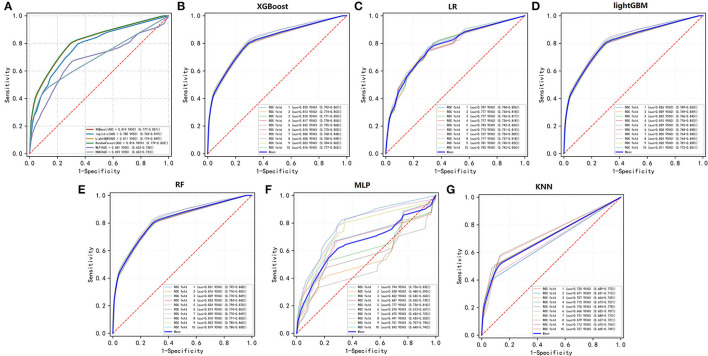
10-fold cross-ROC curves of six ML models in the training cohort; logistic regression (LR), eXtreme Gradient Boosting (XGBoost), light Gradient Boosting Machine (lightGBM), random forest (RF), multilayer perceptron (MLP), and k-nearest neighbor (kNN).

**Figure 4:**
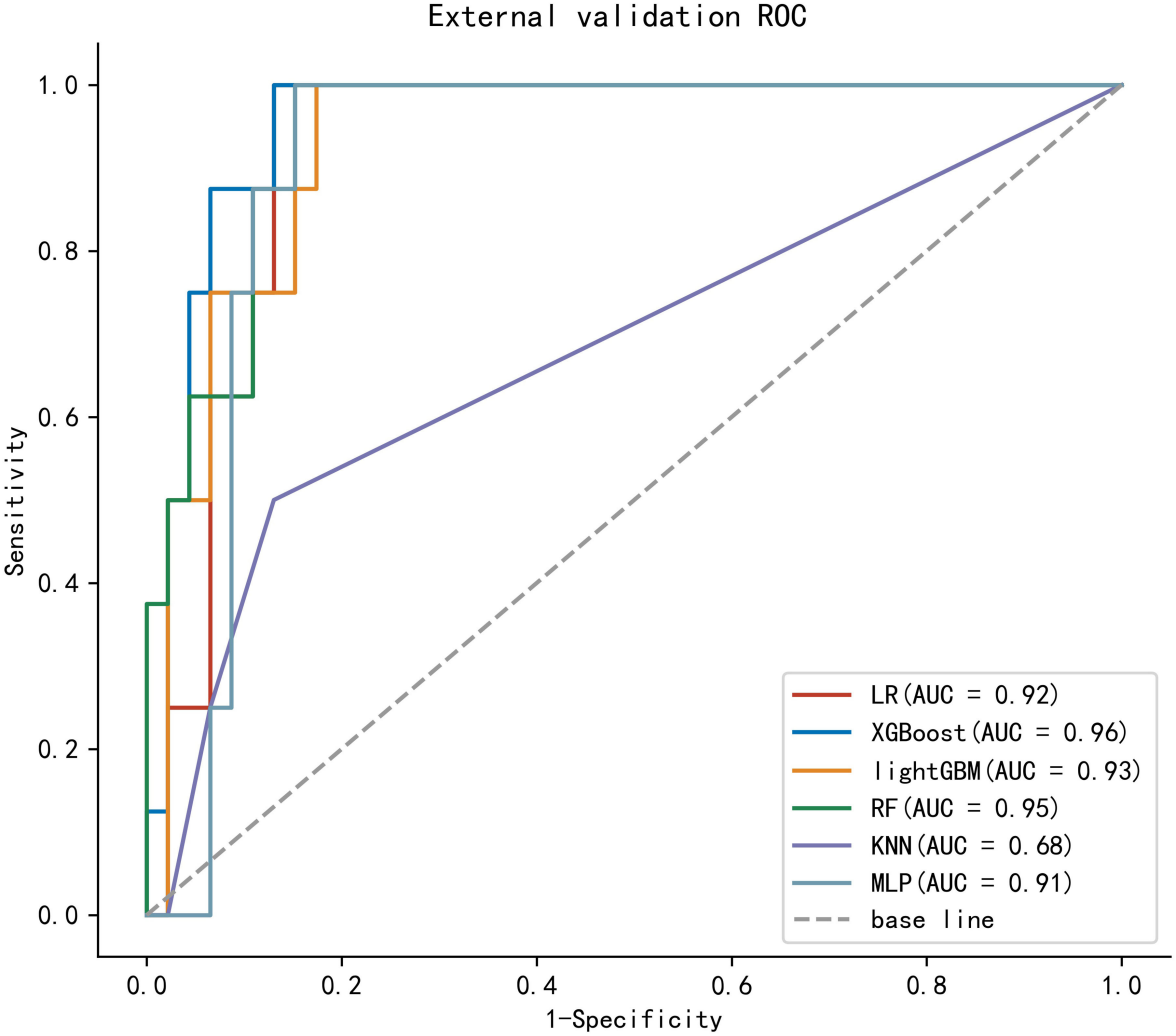
The ROC curves of six models in the external validation cohort.

To compare the predictive efficiency of six ML algorithm models, 10-fold cross-validation was applied in this study (Figure 3). Both the XGBoost model (AUC = 0.814, 95% CI 0.777–0.851) and the RF model (AUC = 0.816, 95% CI 0.779–0.852) performed well in the training cohort. The learning curves of models in the training cohort are shown in Supplementary Figure 2. In external validation, as shown in Figure 4, the XGBoost model (AUC = 0.957, 95% CI 0.904–1.000) showed the best performance in ROC curve analysis among six algorithms, and the RF model also showed great performance (AUC = 0.946, 95% CI 0.886–1.000). Since both the XGBoost model and the RF model were efficient and stable in the training and validation groups, we suggested that both the two algorithmic models can be considered as ideal for predicting the risk of developing M1b stage with patients with GCTC.

### Relative Importance of Variables

**Figure 5:**
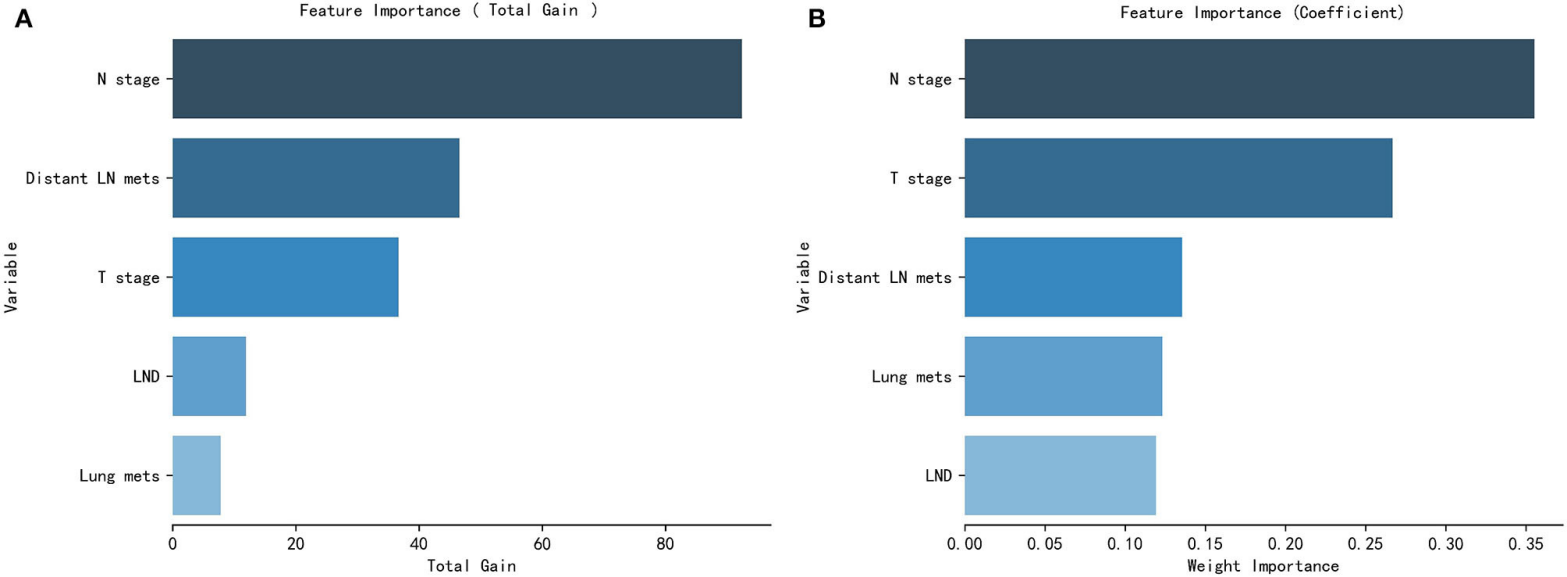
Patients clinical and pathological features' importance of the XGBoost model **(A)** and the RF model **(B)**.

The GCCT patients' clinical feature importance based on the XGBoost and the RF model is shown in Figure 5.

## Discussion

For patients with undetectable metastatic lesions, early application of systemic chemotherapy and combination therapy may improve the prognosis and increase the median survival rate (26). The IGCCCG-related metastatic germ cell testicular cancer prognostic-based staging system (15) is clinically recognized as an effective system. This system showed that for patients with TC who developed metastases, the prognosis for pulmonary metastases was better, whereas patients with non-pulmonary metastases had a poorer prognosis. A recent study also showed that patients with TC who developed organ metastases, such as bone and liver, had over all poor survival and cancer-specific survival (13). Some patients fail to detect metastatic lesions at the first diagnosis or even at the early postoperative review. Some patients with early metastatic GCTC (mGCTC) have subclinical metastases (most common in the retroperitoneum) that are not identified by imaging and are identified and diagnosed as clinical M1 at follow-up after orchiectomy (14, 27). The S-stage is a classification based on serum tumor markers (post-orchiectomy and pre-chemotherapy initiation) and is complementary to the TNM stage. Since the serum half-lives of AFP and β-hCG are 5 to 7 days and 1 to 3 days, respectively, it will take several weeks to return to normal levels (28, 29). These tumor markers not only have prognostic predictive value, but also should be continued during follow-up to assist in determining whether postoperative metastases have occurred (30). The BEP-based (bleomycin, etoposide, and cisplatin) chemotherapy regimen is the standard treatment for metastatic patients with TC (31). A randomized phase III trial showed similar relapse-free survival rates and no significant difference in quality of survival between patients who underwent retroperitoneal lymph node dissection and adjuvant BEP (32). Most patients with GCTC are sensitive to radiotherapy as well (33).

Previous studies have shown that patients with metastases to internal organs other than the lungs have a significantly poor prognosis (13, 15). We confirmed this by obtaining GCTC patients' survival indicators from the SEER database, utilizing the Kaplan–Meier method. Since most patients have no conscious symptoms in the early clinical stage of metastasis, and there is a possibility of missing micrometastases on imaging, the construction of an effective model to predict the risk of stage M1b in patients with GCTC is of great value in clinical application. To the best of our knowledge, this study is the first study to develop an accurate predictive model for predicting the risk of developing the M1b stage in patients with GCTC by incorporating multiple clinical and pathological indicators. In the baseline analysis, we found that the majority of patients received chemotherapy, but only a small percentage of patients received radiotherapy and LND, which is in line with our clinical experience and guideline recommendations. In terms of univariate logistic regression analysis, LND, chemotherapy, T-stage, N-stage, lung metastasis, distant lymph node metastasis, LDH, hCG, AFP, and S-stage were all significantly associated with the occurrence of developing the M1b stage. In the multivariate logistic regression, LND, T-stage, N-stage, lung metastasis, and distant lymph node metastasis were considered significant risk factors. Based on clinical reality, the inclusion of LND in the ML model means that the patient is judged to have an indication for LND by imaging or other assessment modalities preoperatively, rather than receiving LND, which results in an elevated risk of progression to the M1b stage. Unfortunately, in both models of multivariate logistic regression, serum tumor markers were not a predictor of progression to M1b stage in patients with GCTC, which may indicate that serum tumor markers (postoperative LDH, hCG, AFP) are more clinically significant in suggesting metastasis in the lung and distant lymph nodes and have limited predictive value for metastasis in other tissues or organs.

Machine learning is an important branch of AI, which learns the data structure of input data and its intrinsic patterns, selects corresponding learning methods and training methods to construct optimal mathematical models, and continuously adjusts model parameters to seek optimal solutions through mathematical methods to improve generalization ability and effectively prevent the occurrence of overfitting. ML has been widely used in various medical research fields as a powerful algorithm for predictive model building. Compared with traditional statistical methods, ML can better deal with overfitting, unbalanced data distribution and other problems (21, 24, 25). A total of six common ML algorithms were utilized in this study, including LR, XGBoost, lightGBM, RF, MLP, and kNN. The LR algorithm is often thought of as a traditional algorithm, but is essentially a form of machine learning (34). The XGBoost is a ML approach that has the unique ability to integrate missing data quickly and flexibly, as well as to assemble poor prediction models into a more accurate one (35, 36). The RF is a ML classifier that employs multiple trees to train and predict samples. It may be used to reduce training variance and increase integration and generalization (37). The other algorithms included have also shown high prediction accuracy, model stability, and computational efficiency in previous studies (38–40). Integrating the effectiveness and stability of the models in the training and external validation sets, XGBoost and RF were finally identified as two best prediction model algorithms for the risk prediction of progression to M1b in patients with GCTC. We hope to further validate the performance of these two models in the future through collaboration with multicenter medical units, hoping to specify a most efficient algorithmic model and to work with software development experts to develop a mobile program that facilitates clinically friendly applications.

Our study has certain limitations. First, the unavailability of data, including immunohistochemistry, patients' underlying disease, and hematology index, limits the ability to further optimize the ML model, and we hope to incorporate these metrics at a later stage when a multicenter, real-world database is established. Second, S-stage was assessed by the postoperative serum tumor markers we obtained, which may have some human analysis errors because they are not directly available from the database. Meanwhile, the criteria for whether a patient has an indication for adjuvant therapy or LND are inconsistent from one medical institution to another and may be subjected to some errors in practical application. In addition, the practical value of the model obtained based on a predominantly Caucasian database for application in other centers (including China) is unclear due to the inevitable differences in ethnicity and treatment levels in different countries' or regions' validation. Nevertheless, our study is an important step forward in developing a model to predict the risk of developing the M1b stage in patients with GCTC.

## Conclusion

We developed and validated ML algorithms for individualized prediction of the risk of progression to M1b stage in patients with GCTC who underwent orchiectomy by utilizing readily available perioperative patient clinical and pathological data. The ML-based prediction models can identify whether patients are at high risk and may assist the clinician in decision-making.

## Data Availability Statement

The original contributions presented in the study are included in the article/Supplementary Material, further inquiries can be directed to the corresponding authors.

## Ethics Statement

Ethical review and approval was not required for the study on human participants in accordance with the local legislation and institutional requirements. Written informed consent from the participants' legal guardian/next of kin was not required to participate in this study in accordance with the national legislation and the institutional requirements.

## Author Contributions

LD, KW, and CZ contributed to the idea and design. KW, CZ, YZ, and KLRW collected and analyzed the data. LD drew the figures and tables. LD and KW wrote the draft. LD, KW, CZ, YZ, KLRW, WL and JW contributed to manuscript writing and revision. All authors contributed to the article and approved the submitted version.

## Funding

This study was sponsored by the Second Round of Xuzhou Medical Leading Talents Training Project (No. XWRCHT20210027).

## Conflict of Interest

The authors declare that the research was conducted in the absence of any commercial or financial relationships that could be construed as a potential conflict of interest.

## Publisher's Note

All claims expressed in this article are solely those of the authors and do not necessarily represent those of their affiliated organizations, or those of the publisher, the editors and the reviewers. Any product that may be evaluated in this article, or claim that may be made by its manufacturer, is not guaranteed or endorsed by the publisher.

## Supplementary Material

Supplementary Figure 1 Flow chart for patients selection of the SEER database.

Supplementary Figure 2 Learning curves of six ML models in training set and cross-validation set.

Supplementary Figure 3 The definition of S-stage based on the TNM classification for testicular cancer.

Supplementary Table 1 Baseline characteristics of patients with GCTC in the SEER database.

Supplementary Table 2 Original data from SEER database.

Supplementary Table 3 Original data from the Affiliated Hospital of Xuzhou Medical University.
