# Supplementary figures and images for: A Machine Learning Algorithm for Predicting the Risk of Developing to M1b Stage of Patients With Germ Cell Testicular Cancer
Source: Front Public Health. 2022 Jun 29;10:916513. doi: 10.3389/fpubh.2022.916513 (PMC9277219; doi:10.3389/fpubh.2022.916513)

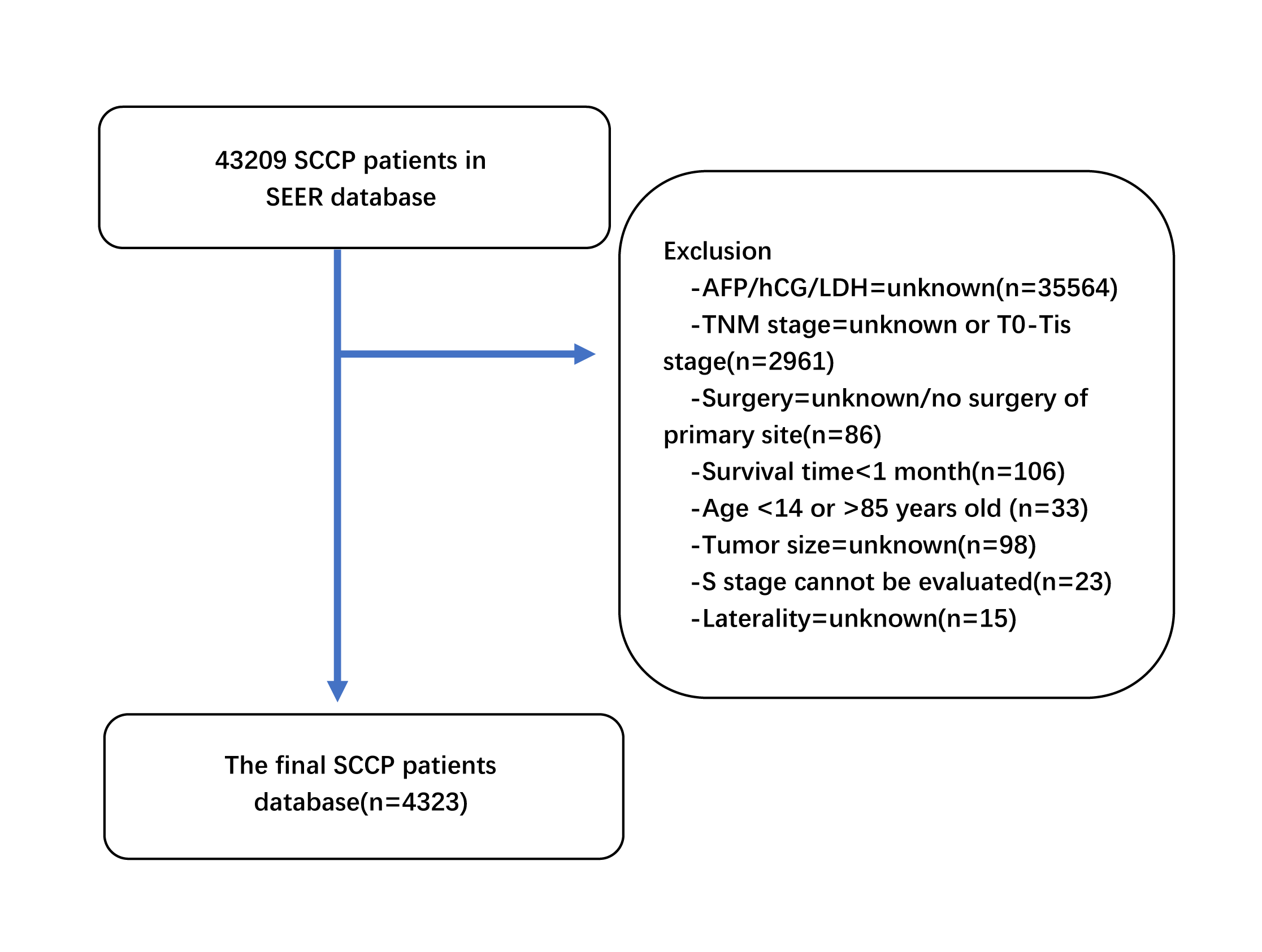

Supplement: Supplementary Figure 1 — Flow chart for patients selection of the SEER database. [file Image_1.TIF]

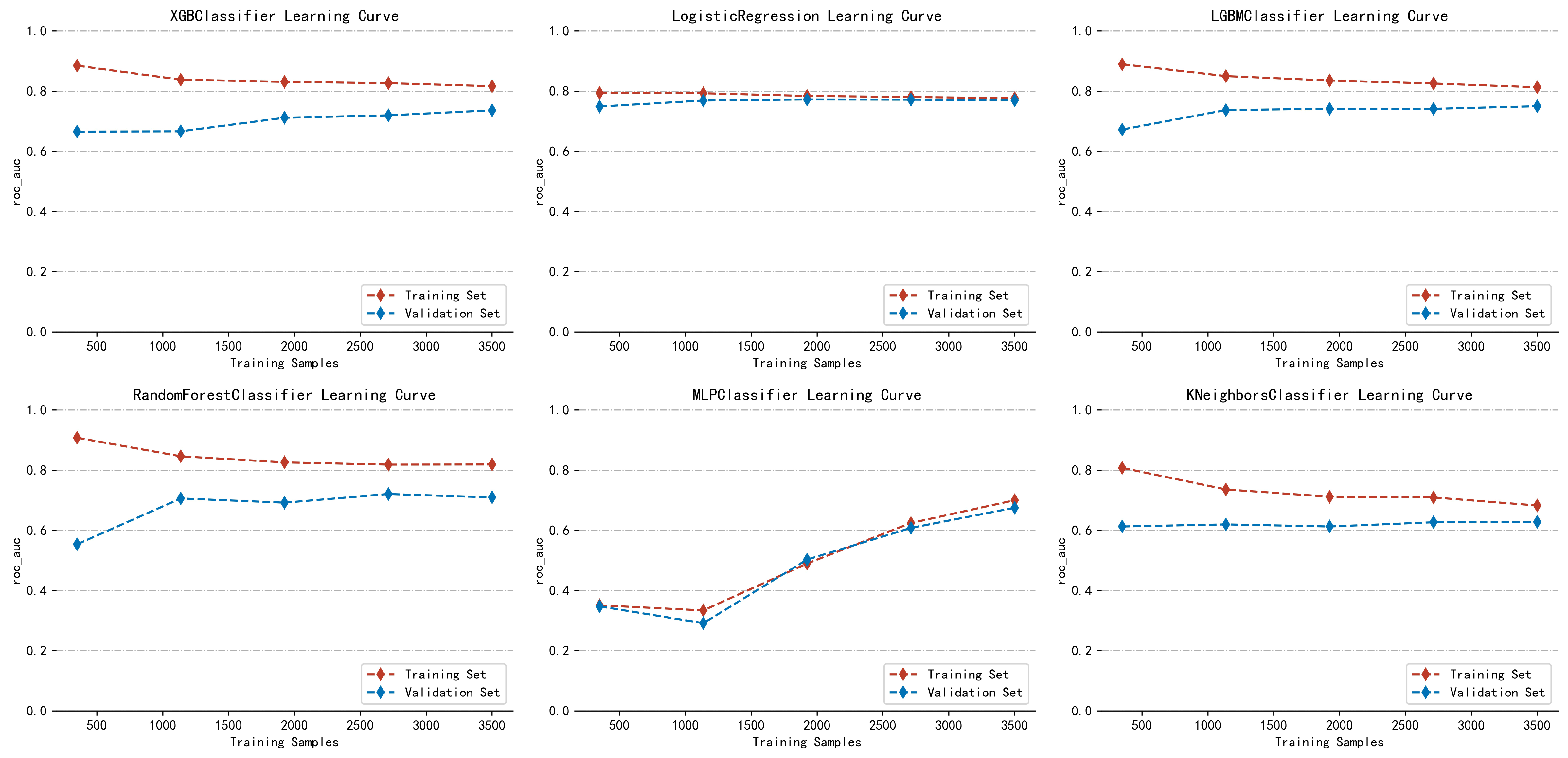

Supplement: Supplementary Figure 2 — Learning curves of six ML models in training set and cross-validation set. [file Image_2.TIF]

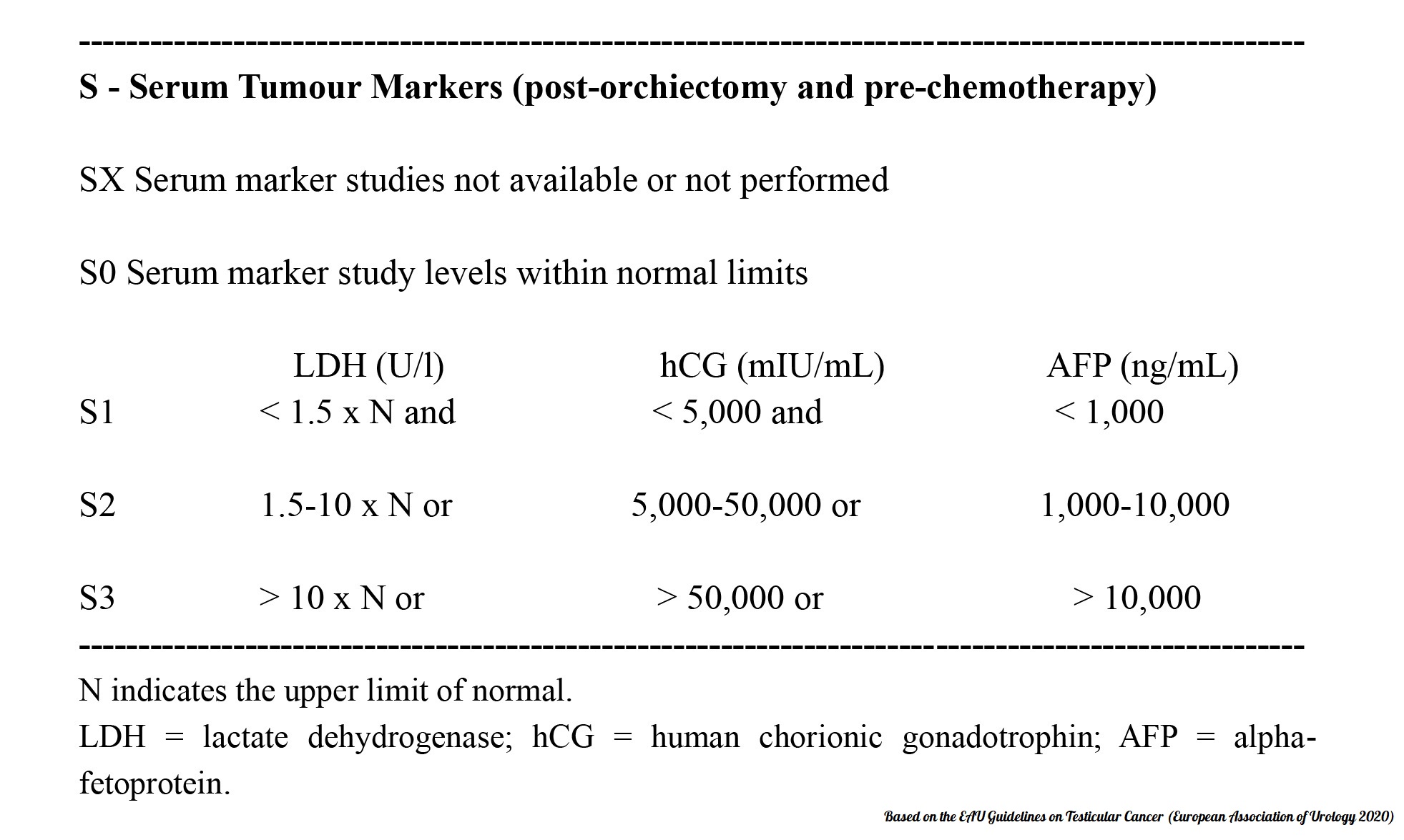

Supplement: Supplementary Figure 3 — The definition of S-stage based on the TNM classification for testicular cancer. [file Image_3.JPEG]
